# Gastrocnemius Medialis Contractile Behavior Is Preserved During 30% Body Weight Supported Gait Training

**DOI:** 10.3389/fspor.2020.614559

**Published:** 2021-01-18

**Authors:** Charlotte Richter, Bjoern Braunstein, Benjamin Staeudle, Julia Attias, Alexander Suess, Tobias Weber, Katya N. Mileva, Joern Rittweger, David A. Green, Kirsten Albracht

**Affiliations:** ^1^Institute of Movement and Neurosciences, German Sport University Cologne, Cologne, Germany; ^2^Department of Medical Engineering and Technomathematics, University of Applied Sciences Aachen, Aachen, Germany; ^3^Institute of Biomechanics and Orthopaedics, German Sport University Cologne, Cologne, Germany; ^4^Centre for Health and Integrative Physiology in Space (CHIPS), Cologne, Germany; ^5^German Research Centre of Elite Sport, Cologne, Germany; ^6^Centre of Human and Applied Physiological Sciences, King‘s College London, London, United Kingdom; ^7^European Astronaut Centre (EAC), European Space Agency, Space Medicine Team (HRE-OM), Cologne, Germany; ^8^KBR GmbH, Cologne, Germany; ^9^School of Applied Sciences, London South Bank University, London, United Kingdom; ^10^Institute of Aerospace Medicine, German Aerospace Center (DLR), Cologne, Germany; ^11^Department of Pediatrics and Adolescent Medicine, University of Cologne, Cologne, Germany; ^12^Institute for Bioengineering, University of Applied Sciences Aachen, Aachen, Germany

**Keywords:** unloading, muscle fascicle behavior, series elastic element behavior, ultrasound imaging, walking, gait, rehabilitation, AlterG

## Abstract

Rehabilitative body weight supported gait training aims at restoring walking function as a key element in activities of daily living. Studies demonstrated reductions in muscle and joint forces, while kinematic gait patterns appear to be preserved with up to 30% weight support. However, the influence of body weight support on muscle architecture, with respect to fascicle and series elastic element behavior is unknown, despite this having potential clinical implications for gait retraining. Eight males (31.9 ± 4.7 years) walked at 75% of the speed at which they typically transition to running, with 0% and 30% body weight support on a lower-body positive pressure treadmill. Gastrocnemius medialis fascicle lengths and pennation angles were measured via ultrasonography. Additionally, joint kinematics were analyzed to determine gastrocnemius medialis muscle–tendon unit lengths, consisting of the muscle's contractile and series elastic elements. Series elastic element length was assessed using a muscle–tendon unit model. Depending on whether data were normally distributed, a paired *t*-test or Wilcoxon signed rank test was performed to determine if body weight supported walking had any effects on joint kinematics and fascicle–series elastic element behavior. Walking with 30% body weight support had no statistically significant effect on joint kinematics and peak series elastic element length. Furthermore, at the time when peak series elastic element length was achieved, and on average across the entire stance phase, muscle–tendon unit length, fascicle length, pennation angle, and fascicle velocity were unchanged with respect to body weight support. In accordance with unchanged gait kinematics, preservation of fascicle–series elastic element behavior was observed during walking with 30% body weight support, which suggests transferability of gait patterns to subsequent unsupported walking.

## Introduction

Orthopedic and neurological rehabilitation regimens often involve patients performing gait training with body weight support (BWS) in an attempt to retrain “natural” walking gait function. Whilst overhead suspension systems are largely employed to promote gait rehabilitation from neurologic disorders (Apte et al., 2018), lower-body positive pressure (LBPP) treadmills are frequently used following orthopedic injuries to re-expose patients to walking whilst bearing progressively greater proportions of their body weight (Quigley et al., 2000; Mishra, 2015). In order to restore gait function, movement patterns should be as similar, and thus transferable to daily activities, as possible albeit with a reduction of lower limb muscle and joint forces (Cutuk et al., 2006). Studies assessing LBPP have demonstrated that whilst ground reaction forces are reduced (Eastlack et al., 2005; Cutuk et al., 2006; Grabowski, 2010), gait kinematics are largely preserved (Apte et al., 2018).

During normal walking, mechanical energy is largely conserved due to the pendulum-like exchange between potential and kinetic energy (Cavagna et al., 2000). Despite this, additional mechanical work by the muscle–tendon unit (MTU) is required to sustain the movement of the body's center of mass. However, walking with BWS reduces the total mechanical energy of the center of mass, and thus presumably requires less force and work from the MTU to vertically support and accelerate the body (Cavagna et al., 2000; Pavei et al., 2015). In fact, significant reductions in the metabolic cost of locomotion have been observed (Farley and McMahon, 1992; Grabowski et al., 2005; Pavei et al., 2015). Furthermore, reductions of knee joint contact forces (Patil et al., 2013), ankle joint moments (Lewek, 2011; Goldberg and Stanhope, 2013; Fischer and Wolf, 2015) ankle joint angular momentum (McGowan et al., 2008) and ankle joint power (Lewek, 2011) have been reported during unloading. Despite reduced kinetic and metabolic requirements for vertical body support and forward acceleration, LBPP (unless BWS is >75%) has been reported to not induce significant differences in spatio-temporal gait parameters such as cadence, stride duration (Grabowski, 2010; Patil et al., 2013) and stride length (Quigley et al., 2000; Cutuk et al., 2006; Patil et al., 2013), nor range of ankle (Quigley et al., 2000; Cutuk et al., 2006) and knee (Eastlack et al., 2005; Cutuk et al., 2006) joint motion. In addition, whilst muscle activity patterns appear unchanged, lower limb muscle activity is reduced during LBPP-treadmill gait (Quigley et al., 2000; Liebenberg et al., 2011; Fischer et al., 2015) with the plantar flexor muscles being particularly susceptible to manipulations of body weight (McGowan et al., 2008). This demonstrates their critical role in human locomotion by providing the majority of the force necessary for vertical body weight support and horizontal propulsion (Neptune et al., 2001; Anderson and Pandy, 2003; McGowan et al., 2008). To gain a better understanding of the plantar flexors' response to different locomotor tasks, ultrasound imaging is a convenient technique to visualize architectural changes, which help to draw conclusions about muscle function.

Ultrasonic visualization of muscle fascicle behavior during locomotion without BWS has not only demonstrated the importance of the storage and release of elastic energy in the Achilles tendon for running and walking (Fukunaga, 2001; Lichtwark et al., 2007), but also that the plantar flexor muscles modulate their behavior depending on gait type, and speed (Farris and Sawicki, 2012). In fact, increased walking speeds have been shown to increase gastrocnemius medialis (GM) shortening velocities (Farris and Sawicki, 2012), and to shorten soleus fascicles (Lai et al., 2015), thereby impairing plantar flexors force generation due to shifting the force–length–velocity relationship toward less favorable contractile conditions (Neptune and Sasaki, 2005; Arnold et al., 2013). However, it is unknown whether walking with BWS modulates fascicle and series elastic element (SEE) behavior to meet the reduced locomotor demands (Richter et al., 2017). Knowledge of any changes in GM's muscle architecture (primarily fascicle length and pennation angle) in addition to fascicle shortening velocity, which affect the force–length–velocity relationships, would facilitate inference of the mechanisms determining mechanical power generation when BWS is applied. Whereas, preservation of fascicle contraction behavior concurrent with preservation of gait kinematics would support the validity of rehabilitative gait training with BWS.

30% BWS is typically recommended for rehabilitative re-introduction to walking and running, due to the preservation of kinematic and spatio-temporal gait parameters (Fischer and Wolf, 2015; Apte et al., 2018) in addition to muscle activation patterns (Neal et al., 2016; Hansen et al., 2017). As during early postoperative rehabilitation patients usually start with recovering their walking function, the present study focuses on walking with BWS. Increasing BWS is known to result in walk-to-run transitions occurring at slower absolute walking speeds (Kram et al., 1997), but similar Froude number, a dimensionless number embedding gait speed, leg lengths and gravitational acceleration (in the present paper expressed as BWS) (Kram et al., 1997; Labini et al., 2011). Thus, to obtain mechanically equivalent speeds (i.e., a similar walking speed relative to the preferred walk-to-run transition speed) at different BWS levels, walking speeds should be adjusted to the same Froude number (Kram et al., 1997; Minetti, 2001; Vaughan and O'Malley, 2005), which requires a reduction in absolute walking speed.

Therefore, the aim of the present study was to determine via ultrasonography GM's fascicle–SEE behavior during walking at mechanically equivalent speeds, namely 75% of the preferred walk-to-run transition speed (PTS), on a LBPP treadmill, with, and without 30% BWS.

It was hypothesized that during walking with BWS (i.e., where forces acting on the SEE are reduced) peak SEE length decreases and is compensated for by longer fascicles and/or smaller pennation angles, rather than by a shorter MTU as ankle and knee joint kinematics are reported to be preserved.

## Materials and Methods

### Participants

Eight healthy male volunteers (mean ± standard deviation: 31.9 ± 4.7 years, 178.4 ± 5.7 cm heights, 94.2 ± 5.6 cm leg lengths, and 73.5 ± 7.3 kg body masses) with treadmill running experience provided informed written consent to participate in this observational study, which received approval from the “Ärztekammer Nordrhein” Ethical Committee of Düsseldorf, Germany. The study was conducted in the Physiology Laboratory of the Institute of Aerospace Medicine at the German Aerospace Center in Cologne, where all participants underwent a standard medical examination. Exclusion criteria included any cardiovascular, musculoskeletal or neurological disorders within the previous 2 years in addition to any lower limb surgery that may affect MTU behavior.

### Study Design and Experimental Protocol

**Figure 1:**
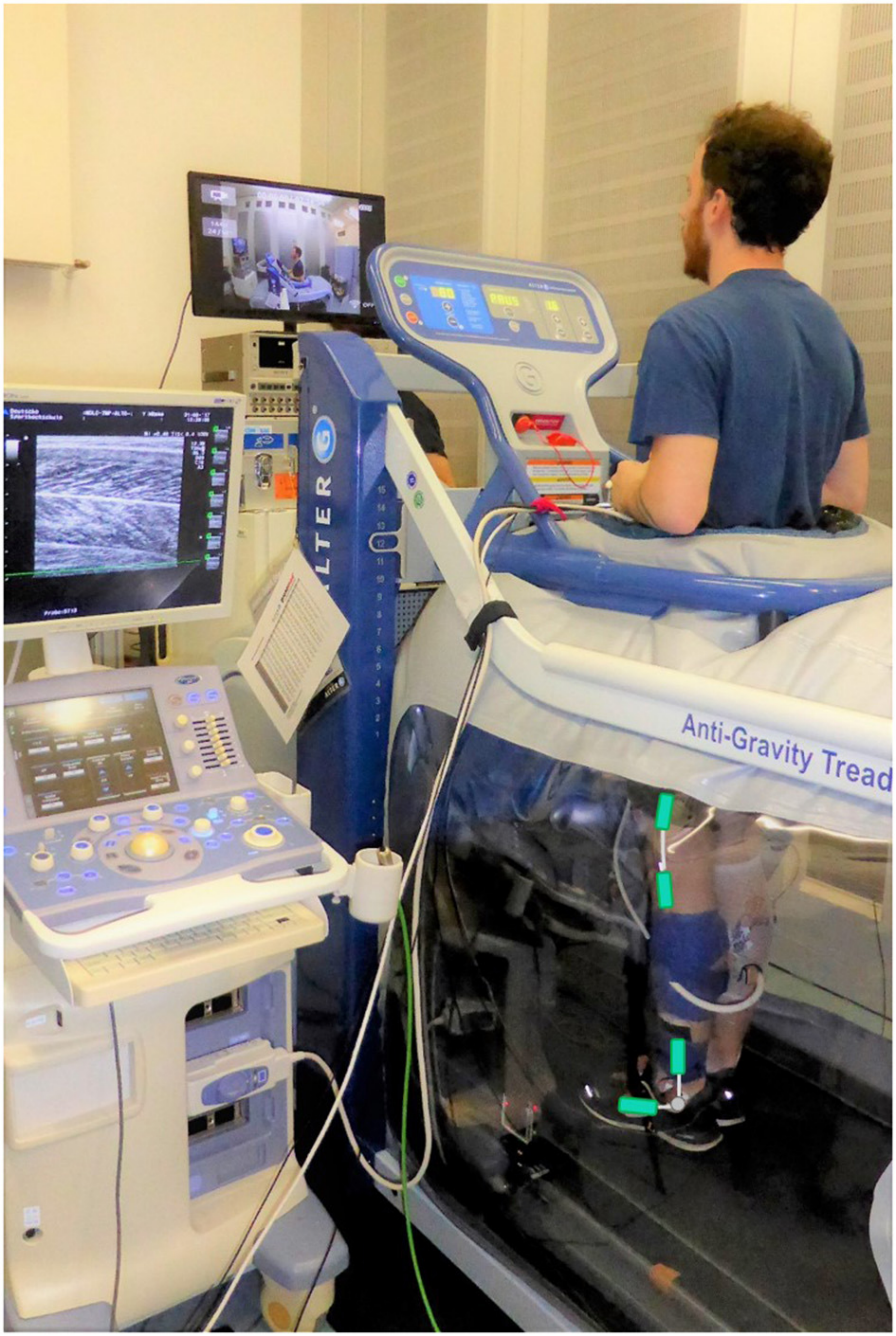
Experimental set-up. Participant walking on the lower-body positive pressure treadmill (the AlterG) with an ultrasound transducer attached to the midbelly of the gastrocnemius medialis muscle and electrogoniometers (added in green to accentuate placement) to measure ankle and knee joint angles.

Participants attended the laboratory on a single occasion and walked on an Anti-Gravity Treadmill (AlterG; AlterG®, M320, Fremont, USA; Figure 1), an LBPP treadmill, with 0% BWS and thereafter with ~30% BWS (recommended load for rehabilitative gait training; Hesse, 2008; Fischer and Wolf, 2015). Before each trial, participants familiarized themselves until they have acclimatized to the BWS level and the predefined walking speed (~4 min). After another 2 min accommodation time given to produce reproducible gait kinematics (Karamanidis et al., 2003) and hence a total warm-up time of ~6 min, which is further required for the Achilles tendon to achieve a relatively stable steady-state behavior (Hawkins et al., 2009), data were collected for 30 s. Blinding of participants was not applicable due to the nature of the experimental set-up.

**Table 1:** Means and standard deviations of kinematic outcome measures while participants walked at 75% of their preferred walk-to-run transition speed with 0% and 30% body weight support.

	**0% BWS**	**30% BWS**	**Differences**	**95% CI**	***P***	**Effect size**
Ground contact time (s)	0.59 ± 0.04	0.62 ± 0.04	0.03 ± 0.03	−0.01 to 0.05	0.078^w^	0.80
Ankle joint angle at touch-down (°)	−6.35 ± 3.26	−7.61 ± 7.74	−1.26 ± 7.17	−7.25 to 4.73	0.635^t^	−0.18
Knee joint angle at touch-down (°)	3.07 ± 5.92	1.24 ± 5.21	−1.84 ± 3.34	−4.63 to 0.95	0.164^t^	−0.55
Ankle joint angle at toe-off (°)	−17.47 ± 7.42	−17.45 ± 7.21	0.02 ± 10.69	−8.92 to 8.95	0.641^w^	<0.01
Knee joint angle at toe-off (°)	47.67 ± 11.38	46.94 ± 7.91	−0.73 ± 10.38	−9.41 to 7.94	0.848^t^	−0.07
Ankle joint range of motion (°)	21.04 ± 5.47	20.88 ± 4.93	−0.16 ± 3.55	−3.12 to 2.81	0.844^w^	−0.04
Knee joint range of motion (°)	45.21± 9.09	45.72 ± 3.77	0.51 ± 7.82	−6.03 to 7.04	0.860^t^	0.06
Ankle dorsiflexion (°)	10.26 ± 3.04	11.82 ± 3.77	1.56 ± 3.15	−1.07 to 4.19	0.204^t^	0.50
Knee flexion (°)	17.82 ± 4.42	15.81 ± 6.49	−2.01 ± 5.65	−6.73 to 2.71	0.347^t^	−0.36
Ankle joint angle at peak SEE length (°)	2.41 ± 3.18	2.72 ± 6.06	0.31 ± 7.91	−6.30 to 6.92	0.742^w^	0.04
Knee joint angle at peak SEE length (°)	9.19 ± 6.83	11.04 ± 4.75	1.85 ± 4.43	−1.86 to 5.55	0.461^w^	0.42

BWS, body weight support; CI, confidence interval; P, result of the paired t-test (t) or Wilcoxon matched-pairs signed rank test (w) indicating a statistically significant effect of body weight support (α = 0.05). n = 8.

Walking speeds were defined as 75% of the preferred walk-to-run transition speed (PTS) expressed as a Froude number (PTS_FR_). PTS_FR_ was estimated by fitting an exponential regression equation $\left(PTS_{FR}\;\left(a\right)=1.183e^{-5.952a}+0.4745\right)$ with a least-squares method (r^2^ = 0.99) to the experimental data of Kram et al. (1997) using the resulting acceleration (*a*) as the independent variable. Hence, for *a* = 0.7 g (*g* = 9.81 m·s^−2^), a PTS_FR_ value of 0.49 was obtained. By accounting for the participants' leg lengths (l), measured from the greater trochanter to the ground, the individual $PTS\left(a\right)=\sqrt{PTS_{FR}\left(a\right)\cdot a\cdot l}$ expressed in meters per second was determined resulting in walking speeds of 1.58 ± 0.05 m·s^−1^ at 0% BWS, and 1.34 ± 0.04 m·s^−1^ at 30% BWS.

The AlterG was enclosed within a sealed height-adjustable chamber, which allowed air pressure to increase inside the chamber and generated an additional vertical buoyant force to produce controlled and stable BWS levels. A seal between the participant and the chamber was created through a neoprene kayak-type skirt that could be zipped into the aperture of the chamber.

### Joint Kinematics

Knee and ankle joint angles were recorded using a twin-axis (Penny and Giles Biometrics Ltd., Blackwood Gwent, UK) and a custom-made potentiometer based electrogoniometer, respectively. The end blocks of the knee electrogoniometer were placed along the leg from the greater trochanter to the lateral femur epicondyle and along the leg from the lateral epicondyle of the femur to the lateral malleolus. The end blocks of the ankle electrogoniometer were placed along the leg from the lateral femur epicondyle to the lateral malleolus and from the lateral malleolus to the most distal end of the fifth metatarsal. Before each walking trial, a reference measurement was taken in the anatomical neutral position to define the 0° joint angles. Data were sampled at a frequency of 1,500 Hz via the TeleMyo 2400 G2 Telemetry System (Noraxon USA., Inc., Scottsdale, USA) and MyoResearch XP software (Master Edition 1.08.16).

### Spatio-Temporal Parameters

To determine gait cycle events and thereby define stance phases of the left leg, participant plantar pressure was measured (83 Hz) via insoles (Novel GmbH, loadsol® version 1.4.60, Munich, Germany). Touchdown and toe-off were automatically detected using a 20 N threshold for 0.1 s via a custom-made script (MATLAB R2018a, MathWorks, Inc., Natick, United States). Insole and electrogoniometer signals were time-synchronized via recording of a rectangular pulse generated by pressing on a custom-made pedal.

### GM Muscle Fascicle Length and Pennation Angle

Real-time B-mode ultrasound (Prosound α7, ALOKA, Tokyo, Japan) captured at 73 Hz using a T-shaped 6-cm linear array transducer (13 MHz) was performed over the midbelly of the left GM muscle. Transducer position was standardized by determining the intersection of the mediolateral and proximodistal midline of the GM and aligning the transducer longitudinally to the fascicles, while transducer movement was minimized by using a custom-made cast, which was secured with elastic Velcro. Ultrasound recordings and electrogoniometer signals were time-synchronized via a rectangular pulse generated by a hand switch, which was recorded synchronously through the electrocardiography channel of the ultrasound and the MyoResearch software. A semi-automatic tracking algorithm (UltraTrack Software, version 4.2; Farris and Lichtwark, 2016) was used to quantify muscle fascicle length (distance between the insertion of the fascicles into the superficial and the deep aponeuroses) and pennation angles (angle between the fascicle and the deep aponeurosis) during the stance phase. Manual correction of the digitized fascicle and the deep aponeurosis, defined as a second fascicle, were performed where appropriate. If the field of view of the transducer was not sufficiently wide to capture the entire fascicle, the missing portion was estimated via manual extrapolation based on the assumption that the fascicle and the aponeurosis extend linearly. Ultrasonography has been frequently used in dynamic conditions (Cronin and Lichtwark, 2013) and has been demonstrated to provide reliable measures of GM fascicle lengths and pennation angles (Aggeloussis et al., 2010; Van Hooren et al., 2020).

### SEE and MTU Lengths

Series elastic element length was estimated using an MTU model by subtracting muscle fascicle lengths multiplied by the cosine of their pennation angles from the MTU lengths (Fukunaga, 2001). Muscle–tendon unit length was calculated via a linear regression equation (Hawkins and Hull, 1990), using participant's shank length data (the distance from the lateral malleolus to the lateral femur epicondyle) in addition to recorded knee and ankle joint angles.

### Data Processing

For each participant, and each outcome, eight consecutive stance phases (touchdown to toe-off) of the left foot per condition were analyzed and averaged using custom-made scripts (MATLAB R2018a, MathWorks, Inc., Natick, United States). Prior to being resampled to 101 data points per stance phase (to represent data as a percentage), fascicle lengths and pennation angles were smoothed with a five-point moving average. Electrogoniometer signals were smoothed with a fifth-order Butterworth low-pass filter, and a 10-Hz cut-off frequency. Fascicle velocity was calculated as the time derivative of its length using the central difference method (Robertson et al., 2013).

Based on the ultrasound and joint-angle recordings SEE length, MTU length, fascicle length, pennation angle, and fascicle velocity were determined at the time when peak SEE length was achieved, and thus force acting on the SEE is presumably at its greatest. Furthermore, average values across the stance phase were determined. Overall fascicle shortening was calculated by subtracting the minimum from maximum fascicle length. Knee and ankle joint range of motion were defined as the delta between their respective minimum and maximum joint angles. Additionally, the difference in knee and ankle joint angles between touchdown to the time of first local maximum and maximum dorsiflexion, were defined as knee flexion and ankle dorsiflexion, respectively. Knee and Ankle joint angles at touchdown and toe-off as well as ground-contact times were determined. To estimate the level of BWS achieved by applying LBPP, average plantar forces over the stance phase were determined and expressed as percentage of the average plantar forces when walking without BWS.

### Statistical Analysis

Distribution normality was assessed using the Shapiro–Wilk normality test. If normally distributed, a two-tailed paired *t*-test was performed, whereas if not, a non-parametric Wilcoxon (matched-pairs) signed rank test was used to compare conditions (30 vs. 0% BWS). All tests were performed in GraphPad Prism (v 7.04) with a significance level of α = 0.05. Effect Sizes (d_z_) were calculated using the G^*^Power software version 3.1.9.4 (Faul et al., 2007). Thresholds of 0.2, 0.5 and 0.8 were defined as small, moderate and large effects between the two comparison groups (Cohen, 1988).

## Results

Participants walking with 30% BWS generated significantly lower average plantar forces (−194 ± 32 N, *P* < 0.001, d_z_ = −6.07) corresponding to 68 ± 4% of the average plantar forces when walking without BWS which did not differ significantly from the target of 70% (*P* = 0.223). Ground-contact times were 0.03 ± 0.03 s longer when walking with 30% BWS, however, the effect was statistically not significant (*P* = 0.078, *d*_*z*_ = 0.80) (Table 1).

**Figure 2:**
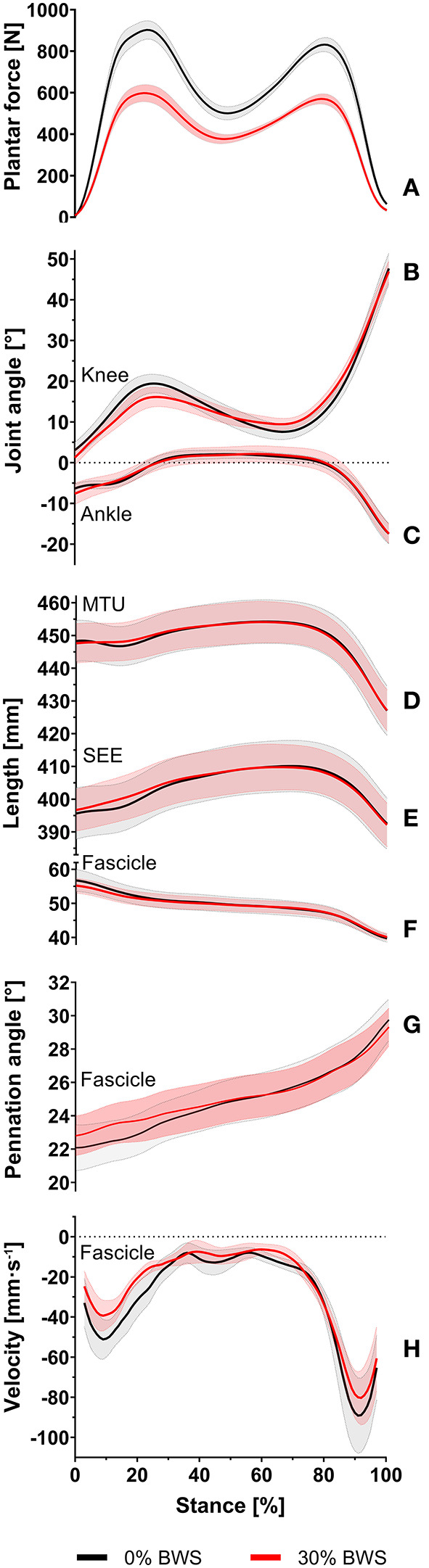
Sample average and standard error of plantar forces **(A)**, knee joint angle **(B)**, ankle joint angle **(C)**, muscle-tendon unit length **(D)**, series elastic element length **(E)**, fascicle length **(F)**, pennation angle **(G)**, and fascicle velocity **(H)** for participants walking at 75% of their preferred walk-to-run transition speed with 0% body weight support (black line) and 30% body weight support (red line) during the entire stance phase. The solid lines represent the sample average, and the corresponding shaded areas represent the standard error of measurement.

Figure 2 presents the averages and standard errors of joint angles and muscle–SEE outcomes time normalized to a single stance phase for participants walking with 0 and 30% BWS.

No statistically significant differences in knee and ankle joint angles at touchdown (*P* = 0.164, *d*_*z*_ = −0.55; *P* = 0.635, *d*_*z*_ = −0.18), at toe-off (*P* = 0.848, *d*_*z*_ = −0.07; *P* = 0.641, *d*_*z*_ < 0.01) and at the time of the peak SEE length (*P* = 0.461, *d*_*z*_ = 0.42; *P* = 0.742, *d*_*z*_ = 0.04) were observed (Table 1). Furthermore, knee and ankle joint range of motion (*P* = 0.860, *d*_*z*_ = 0.06; *P* = 0.844, *d*_*z*_ = −0.04), knee flexion (*P* = 0.347, *d*_*z*_ = −0.36) and ankle dorsiflexion (*P* = 0.204, *d*_*z*_ = 0.50) were unaffected by walking with 30% BWS (Table 1).

**Figure 3:**
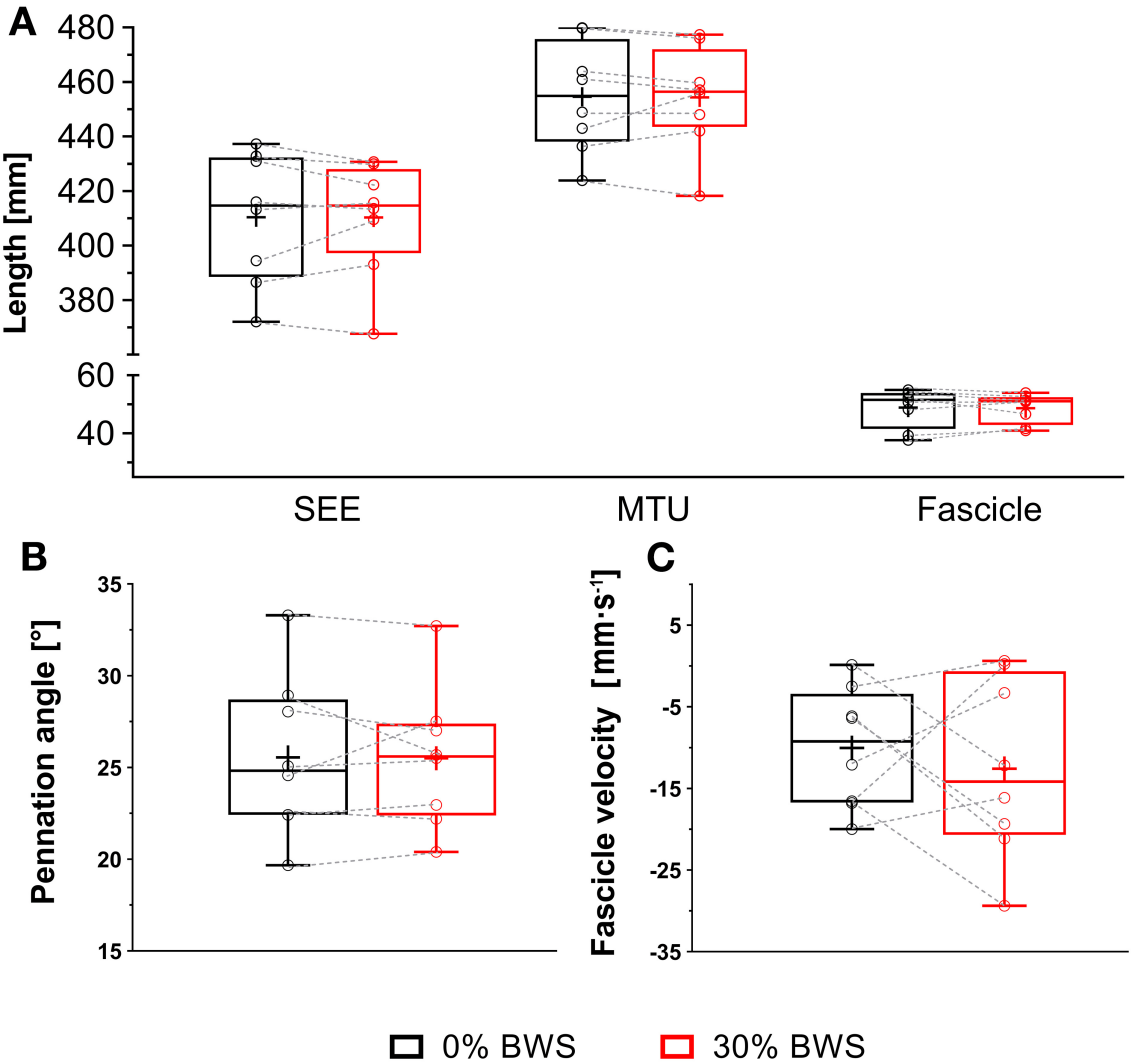
Series elastic element length **(A)**, muscle-tendon unit length **(A)**, fascicle length **(A)**, pennation angle **(B)**, and fascicle velocity **(C)** at the time of the peak series elastic element length as presented as boxplots for participants walking without body weight support (black box) and 30% body weight support (red box). The lower and upper parts of the box represent the first and third quartile, respectively. The length of the whisker represent the minimum and maximum values. The horizontal line in the box represents the statistical median of the sample; +, the mean of the sample; °, individual data points.

Walking with 30% BWS had no effect on peak SEE length (*P* = 0.976, *d*_*z*_ = −0.01) (Figure 3A). Furthermore, at the time when peak SEE length was reached, no statistically significant differences from 0% BWS were observed for MTU length (*P* = 0.641, *d*_*z*_= −0.04), fascicle length (*P* = 0.890, *d*_*z*_ = −0.05), pennation angle (*P* = 0.945, *d*_*z*_ = −0.03) and fascicle velocity (*P* = 0.576, *d*_*z*_ = −0.21) (Figures 3A–C).

No statistically significant differences of the average values across the entire stance phase were also observed for SEE length (*P* = 0.945, *d*_*z*_ = 0.05), MTU length (*P* = 0.641, *d*_*z*_ = 0.01), fascicle length (*P* = 0.790, *d*_*z*_ = −0.10), pennation angle (*P* = 0.641, *d*_*z*_ = 0.16) and fascicle velocity (*P* = 0.148, *d*_*z*_ = 0.51) between 0 and 30% BWS walking (Table 2). Furthermore, overall fascicle shortening did not differ between conditions (*P* = 0.313, *d*_*z*_ = −0.43) (Table 2).

**Table 2:** Means and standard deviations of gastrocnemius medialis muscle and SEE outcome measures while participants walked at 75% of their preferred walk-to-run transition speed with 0 and 30% body weight support.

	**0% BWS**	**30% BWS**	**Differences**	**95% CI**	***P***	**Effect size**
Average SEE length (mm)	404.19 ± 23.40	404.62 ± 20.74	0.42 ± 7.70	−6.02 to 6.86	0.945^w^	0.05
Average MTU length (mm)	449.11 ± 19.80	449.18 ± 18.27	0.07 ± 6.24	−5.15 to 5.28	0.641^w^	0.01
Average fascicle length (mm)	49.44 ± 6.48	49.18 ± 5.28	−0.25 ± 2.58	−2.41 to 1.90	0.790^t^	−0.10
Average pennation angle (°)	24.92 ± 3.93	25.16 ± 3.84	0.24 ± 1.46	−0.98 to 1.46	0.641^w^	0.16
Average fascicle velocity (mm·s^−1^)	−31.03 ± 16.18	−26.01 ± 8.72	5.02 ± 9.91	−3.27 to 13.31	0.148^w^	0.51
Overall fascicle shortening (mm)	17.23 ± 7.54	15.12 ± 4.64	−2.10 ± 4.91	−6.20 to 2.00	0.313^w^	−0.43

BWS, body weight support; CI, confidence interval; P result of the paired t-test (t) or Wilcoxon matched-pairs signed rank test (w) indicating a statistically significant effect of body weight support (α = 0.05). n = 8.

## Discussion

### Effects of Walking With 30% BWS on Contractile and Series Elastic Element Behavior

During the walking trials, participants were successfully unloaded by 30% of their body weight, as the average plantar forces actually achieved by inducing LBPP did not differ significantly from the target average plantar forces. The main findings were that walking with 30% BWS did not significantly affect joint kinematics. Furthermore, in contrast to the hypotheses, walking with 30% BWS induced no statistically significant differences from 0% BWS in peak SEE length as well as MTU length, fascicle length, and pennation angle neither at the time of the peak SEE length, nor on average across the stance phase. Also, in contrast with the hypotheses, no statistically significant effect of 30% BWS was found on fascicle shortening velocity at the time of the peak SEE length, nor on average across stance, despite a reduction in absolute walking speed (albeit same Froude number). These findings are further supported by the overall small effect sizes.

Previous studies and simulation models have shown that the GM force-length-velocity behavior shifts with gait type and speed to meet the varying locomotor demands (Farris and Sawicki, 2012; Arnold et al., 2013). However, in the present study, fascicle length and pennation angle were unchanged when walking with 30% BWS, which implies that the GM remains operating on a similar part of the force–length relationship, thereby preserving its force generation ability (Arnold et al., 2013). Moreover, GM fascicle velocity has been reported to decrease with decreasing walking speed, thereby increasing GM's force generation ability (Farris and Sawicki, 2012; Arnold et al., 2013). In fact, in the present study average fascicle velocity was 5.0 ± 10 mm·s^−1^ slower when walking at a slightly slower speed at 30 vs. 0% BWS (−0.24 m·s^−1^) reaching a moderate effect (*d*_*z*_ = 0.51), however, high variability may have contributed to it failing to reach statistical significance.

It has been reported that the ankle plantarflexion moment decreases with increasing BWS (Lewek, 2011; Goldberg and Stanhope, 2013; Fischer and Wolf, 2015). In fact, the present results suggests a reduction in average plantar force by almost 200 N whilst ankle joint kinematics were largely preserved when walking with 30% BWS suggesting that ankle joint moment was reduced. Interestingly, this did not affect peak SEE length, which incorporates the length of the free tendon and aponeurosis. As aponeurosis stiffness varies upon contractile conditions (e.g., reduced muscle activity results in lower orthogonal muscle expansion linked to lower transverse strain) (Azizi and Roberts, 2009), SEE length can remain similar despite a reduction in ankle joint moment.

However, as in the present observational study MTU interaction was only modeled for the GM, which accounts for a modest fraction (~17%) of the physiologic cross-sectional area of the plantar flexor muscles (Ward et al., 2009), changes that influence the ankle joint moment might not be fully reflected. Furthermore, joint moments were not determined and SEE length was not measured directly but estimated using an MTU model. Thus, if tuning of the mechanical properties of the SEE actually causes preserved SEE and fascicle kinematics warrants further study.

### Implications for Rehabilitative Body Weight Supported Gait Training

Maintenance of joint kinematics and GM behavior may facilitate rehabilitative gait training by preserving “natural” movement patterns, despite joint loads and related pain being reduced (Eastlack et al., 2005; Cutuk et al., 2006). Preserved fascicle's operating range suggests that the stimuli exerted on the muscle remain the same and thus help to maintain optimum fascicle length for force production, which is key for locomotor recovery. Furthermore, the maintenance of SEE strain, as possibly achieved by an increased aponeurosis strain, might help to prevent degeneration and maintain function of the aponeurosis despite external unloading. Patients who may benefit from LBPP gait training during their early postoperative rehabilitation include not only those with tendon, ligament and meniscus repairs but also joint replacements or fractures (Eastlack et al., 2005). However, the increased aponeurosis strain, which is required to compensate for the decreased free tendon strain (and thus to maintain SEE strain), could pose a potential risk to patients after Achilles tendon rupture if the rupture does not exclusively affect the free tendon. Therefore, BWS rehabilitation should be individualized to the specific pathological characteristics of patients, depending on the impaired biological tissues that require unloading, e.g., rehabilitation after total knee arthroplasty vs. ankle or Achilles tendon injury. Based on the current findings, further studies including different patient groups are required.

The present data, are not only in agreement with a recent systematic review, which concluded that spatio-temporal and kinematic gait parameters can be preserved with up to 30% BWS (Apte et al., 2018), but extends this view to preserved muscle–SEE mechanics. In fact, healthy individuals appear able to retain normal walking kinematics even when unloaded by up 50% BWS (Van Hedel et al., 2006; Awai et al., 2017). The absence of any effects when BWS was increased from 0 to 30% suggests that the modulation of fascicle–SEE behavior does not develop linearly with increasing BWS but is determined by a certain threshold, however if this threshold is below or above 50% BWS remains to be determined. Additionally, if non-LBPP BWS systems, such as overhead suspension harnesses, therapist-assisted waist belts or robotic-assisted gait-training devices, are also able to preserve GM behavior warrants further study. Nevertheless, the present observational study supports the recommendation (Fischer and Wolf, 2015) for LBPP-induced 30% BWS in rehabilitative gait training. Finally, it should be noted that walking speed was intentionally reduced with increasing BWS via the adjustment to the same Froude number to obtain mechanically equivalent walking speeds (Vaughan and O'Malley, 2005). Thus, the observation that the neural system appears to largely preserve GM overall contraction behavior in addition to joint kinematics suggests that the approach of producing comparable gait patterns across the different walking conditions was successful and should be considered for future gait rehabilitation.

## Conclusions

This is the first study to examine *in vivo* GM fascicle–SEE behavior during walking at 30% BWS, frequently employed in gait rehabilitation, at 75% PTS on an LBPP treadmill. The present findings reveal that during walking with 30% BWS fascicle–SEE behavior was largely preserved, in contrast to the hypothesis. Thus, the present study not only supports the contention made in previous studies that walking with the recommended therapeutic dose of 30% BWS largely retains spatio-temporal and joint kinematic characteristics but extends this to GM fascicle and SEE mechanics. This may be advantageous during rehabilitative gait training with BWS as it indicates transferability of gait patterns to subsequent unsupported walking.

## Data Availability Statement

The original contributions presented in the study are included in the article/supplementary materials, further inquiries can be directed to the corresponding author/s.

## Ethics Statement

The study involving human participants were reviewed and approved by Ärztekammer Nordrhein Ethical Committee of Düsseldorf, Germany. The patients/participants provided their written informed consent to participate in this study. Written informed consent was obtained from the individual(s) for the publication of any potentially identifiable images or data included in this article.

## Author Contributions

BB, TW, and DG conceptualized research. BB, JA, TW, KM, JR, DG, and KA designed research. CR, BB, BS, JA, and AS acquired data. CR, BB, BS, AS, and KA analyzed data. CR, BB, JR, DG, and KA interpreted data. CR and DG drafted manuscript. BB, JA, TW, KM, JR, DG, and KA revised manuscript. All authors approved manuscript.

## Conflict of Interest

DG and TW are employed by KBR on behalf of the European Space Agency. The remaining authors declare that the research was conducted in the absence of any commercial or financial relationships that could be construed as a potential conflict of interest.

## Abbreviations

AlterG: AlterG® Anti-Gravity TreadmillTM
BWS: body weight support
g: earth's gravitational acceleration
GM: gastrocnemius medialis
LBPP: lower-body positive pressure
MTU: muscle–tendon unit
PTS: preferred walk-to-run transition speed
SEE: series elastic element.
